# Impact of an End-User-Designed Education Bundle for Electronic Health Records System Education of House Officers

**DOI:** 10.7759/cureus.63102

**Published:** 2024-06-25

**Authors:** Guan Lin Lee, Albert Teo, Audrey Ho, Michael Chu, Yi-Shi Kelly Chng, Shalini Sri Kumaran

**Affiliations:** 1 Department of Internal Medicine, Singapore General Hospital, Singapore, SGP; 2 Eunos Polyclinic, Singhealth Polyclinics, Singapore, SGP; 3 Singhealth-Duke NUS Family Medicine Academic Clinical Program, Singhealth, Singapore, SGP

**Keywords:** work productivity, electronic health record system, tiktok, electronic health records, medical education

## Abstract

Introduction

The benefits of Electronic Health Records (EHR) use in clinical care are well documented. However, without proper education and training on EHR systems, clinicians may face challenges in utilizing these technological tools effectively. Suboptimal usage of EHR systems can affect productivity. This study assesses the effectiveness of an end-user-designed education bundle as a supplement to existing training in EHR training for house officers. Additionally, it evaluates the effectiveness of using non-conventional teaching modalities (i.e., short TikTok-style videos) to see how effective and accepted it was in comparison to traditional educational material.

Methods

A single-armed pre-post-study design consisting of 36 house officers was employed to evaluate the effectiveness of the intervention bundle. The bundle consists of a series of EHR tips and tricks as identified by experienced senior medical officers. The three components of the bundle are a handbook with consolidated tips and tricks, a long-form lecture video, and a series of TikTok-style videos. Distribution was done through healthcare collaborative platforms such as TigerConnect™ (Los Angeles, USA) and email.

Results

Participants found that the inclusion of our supplementary education bundle results in more effective training for EHR usage, with mean effectiveness with and without the educational bundle being 7.77 and 6.44, respectively (p < 0.001). There were also significant improvements in ease of finding information (7.67 vs 7.14, p = 0.016), performing general functions (7.50 vs 6.89, p = 0.0050), and overall efficiency (7.39 vs 6.92, p = 0.022). We also found TikTok-style videos were non-inferior to more traditional forms of education such as a handbook and traditional long-form lecture videos (p = 0.250).

Conclusion

An end-user-driven education bundle focusing on high-yield, advanced functions may be useful in enhancing the overall EHR system experience for junior doctors. Of note, TikTok-style videos may be no less effective than traditional methods of EHR teaching.

## Introduction

The benefits of using Electronic Health Records (EHR) in healthcare delivery and patient care have been well studied in the literature, with improved efficiency in information management, clinical decision-making, and patient safety being just some of the advantages conferred [1,2]. As electronic information storage, it allows clinicians to search quickly for information, organize data efficiently, and access patient medical records more easily over different visits or even across different healthcare institutions. This last point results in a continuous and seamless provision of care [3] and is of particular importance in modern medicine. The ability to access clinical information to support clinical decisions is paramount with increasing life spans and chronic disease burden in our patients against a backdrop of improved medical knowledge, increased investigation options, and a variety of treatments [4].

However, without proper education and training on EHR systems, clinicians may face challenges in utilizing these technological tools effectively. A study by Jeyakumar et al. [5] emphasized the significance of good EHR education, as it enables clinicians to navigate, interpret, and input data accurately, ensuring the integrity and usability of patient information. Existing literature has also suggested that suboptimal satisfaction with the difficulties of EHR use plays a significant role in physician burnout, an area that the realm of medical education can try to address [6,7]. Improved mastery of the EHR system also results in improved physician productivity [8], which can only be beneficial in high patient load settings (e.g., aging populations, pandemic settings).

A systematic review by Samadbeik et al. [9] identified five main methods of education. They are namely, one-on-one training, peer-coach training, classroom training (CRT), computer-based training (CBT), and blended training - which is a combination of CRT and CBT, with CBT alone accounting for 45% of EHR training. Possible reasons for CBT being the preferred option for the majority of institutions include standardization, scalability, and accessibility [10]. Yet, how best to design CBT is not clear.

In our institution, healthcare professionals utilize Sunrise Clinical Manager (SCM), which is an EHR system built by Allscripts. Education is largely done through CBT, an interactive online course that brings learners through various functions of the EHR system. Our education bundle served to supplement existing training material by identifying a series of high-yield, advanced functions that collectively make the EHR experience more efficient. In addition, we also wanted to pilot the use of less conventional teaching modalities (i.e., short TikTok-style videos) to see how effective and accepted it was in comparison to traditional educational material.

## Materials and methods

Study design

The focus of this paper is a single-armed pre-post study design employed to evaluate the effectiveness of an intervention bundle in House Officers (HO). All study participants were HOs who rotated through the Department of Internal Medicine (DIM), no more than one month after the start of their posting. Data were collected between February 15, 2023 to March 15, 2023 and primary study participants consisted of 36 HO. For this particular arm, the decision was made for participants to act as their own control group, comparing responses pre and post-intervention bundle.

This study design was decided upon based on two reasons. Firstly, the risk of exposure effects, if a separate control arm within the same rotation of HOs was chosen, was nontrivial due to the nature of the intervention material. Given the entertainment value of the education material and its electronic nature, the authors thought that there was a good likelihood of achieving some degree of virality through dissemination channels, and hence difficult to ensure no exposure to a separate control arm within the institution. If exposure happens, the impact of the intervention bundle may be underestimated. Secondly, the authors considered but did not proceed with a separate rotation of HOs because of concerns regarding comparability between different HO rotations, who would have different demographic and training backgrounds such that result interpretation may prove tricky. Adopting a study design where participants acted as their own controls would overcome this point.

Separately, a prior population of 36 HOs (secondary arm) was evaluated from October 4, 2022 to October 31, 2022 to assess for baseline SCM task efficiency; they were not exposed to the intervention bundle and served as a proxy ‘pre-intervention’ population for our primary study group. This was done to limit any practice effects that may be seen within our primary study participants when it comes to the evaluation of SCM task function. The four-month interval between data collection was due to HO rotational change. 

Intervention bundle

Contents of the intervention bundle were created by our team of five senior Medical Officers (MO) with between four to six years of ground experience and still actively using the EHR system for daily clinical work. The team was supervised by a DIM Consultant. The intervention bundle consisted of a series of tips and tricks that were essential in improving SCM efficiency but were not widely known by HOs. This series of tips and tricks was consolidated through the collective experience of the team. Basic understanding and usage of SCM by HOs were expected, given prior exposure to institution CBT, and the aim of the intervention bundle was to improve HO efficiency and satisfaction with SCM.

The intervention bundle consisted of three components. Firstly, a 37-page handbook with consolidated tips and tricks (Appendix A) was made into an electronic PDF document for ease of accessibility to allow for quick reference even while working. Secondly, a long-form video of approximately 35 minutes (Video 1) was prepared using the same collection of high-yield tips and tricks. Thirdly, the team voted and consolidated the top 10 tips and tricks. The content was then translated into 10 short-form TikTok-style videos ranging from five to 33 seconds (Videos 2, 3 for sample material). The videos were inspired by existing trends on TikTok videos, and these were used as a model to deliver concise and impactful information in an entertaining fashion. The intervention bundle was distributed through healthcare collaborative platforms such as TigerConnect™ and work email when the HOs started their posting. Two TikTok-style videos were released each day over five days. These channels were selected as they were official work communication tools that are readily accessible by all HOs at their workplace.

**Video 1:** Lecture style video (Wizlearn)

**Video 2:** TikTok-style video - sample 1

**Video 3:** TikTok-style video - sample 2

As this study was a quality-improvement project, a formal application for ethical approval does not apply according to institution guidelines.

Data collection

Data for the primary study were collected using self-administered questionnaires (Appendix B). For the self-administered questionnaires, participants were tasked to rate the effectiveness of existing training material after the inclusion of the supplementary education bundle. They were also asked to rate (1) the ease of finding information in SCM, (2) the ease of performing functions within SCM, (3) perceived efficiency in using SCM, and (4) perceived satisfaction in using SCM. All four parameters were measured on a 1-10 scale, and participants were asked to rate before and after the inclusion of education bundle. Google Survey was employed to collect survey data. Study group data collected included education background and prior experience of SCM use. Qualitative data were also collected at the end of the survey to assess for overall consensus on the interventions being employed, and areas for improvement.

SCM task competency was additionally assessed using specially designed tests (SDTs) to assess HOs on SCM functions. SDTs were designed with an aim to objectively assess a HO's competency in 12 pre-selected basic SCM tasks. Tasks tested ranged from finding and displaying relevant information to customization of SCM (Appendix C). SDTs were administered and assessed by team members to HOs. Briefly, 12 tasks were randomly pre-selected based on the consolidated list of tips and tricks. Printouts for test administrators were done to ensure standardization of each task to each participant. Each successful task was given a score of one, with a maximum score of 12 achievable. A task is only considered successful if the participating HO is aware of the tested task and able to complete it within a reasonable amount of time independently.

Statistical analysis

The primary endpoint was to evaluate for improved effectiveness of SCM training material. The secondary endpoint was to evaluate for change in satisfaction and efficiency of SCM usage. Significance was determined using paired student’s t-test for comparisons of median scores pre and post intervention. Significance of the intervention bundle on participant SCM task efficiency was analyzed with two-sample T-test as two different populations of HO were used (as described above). Repeated measure ANOVA was performed to compare if the three different components (Handbook, Wizlearn-styled and TikTok-style videos) of our intervention bundle significantly differed from one another. Data were analyzed using STATA (StataCorp., College Station, TX) and de-identified for all statistical analyses.

## Results

Study participants

There was a total of 72 study participants. All participants were medical graduates, with 78.8% (n=28) in the primary arm of the study being locally trained, while 88.9% (n=32) in the secondary arm were locally trained (Table 1). Locally trained participants were from the Yong Loo Lin (YLL) School of Medicine or the Lee Kong Chian (LKC) School of Medicine. The authors compared the proportions of participants from a local medical school, and the proportion of participants with prior experience with SCM usage (Table 2), as these two factors are likely to play a role in HOs being more efficient with EHR use and lead to comparability issues between the two arms. However, it was found that there was no statistical significance between the primary and secondary arm participants in terms of locality (p = 0.206). Prior experience in SCM was also not significantly different between the two groups (p = 0.909).

**Table 1:** Comparisons between the locality of training between participants in primary and secondary arm *Pearson Chi-Squared Test

Locality of Training	Primary Arm (n = 36)		Secondary Arm (n = 36)		P-value
	n	%	n	%	
Locally Trained	28	78.8	32	88.9	0.206^*^

**Table 2:** Comparisons of mean exposure to Sunrise Clinical Manager (SCM) between participants in primary and secondary arm †2-Sample T-Test

Exposure to SCM	Primary Arm (n = 36 )	Secondary Arm (n = 36)	P-value
	Mean ± SD	Mean ± SD	
Mean exposure to SCM prior to posting (months)	6.444 ± 2.660	5.722 ± 1.814	0.908^†^

Quantitative results

Participants found the inclusion of a supplementary education bundle to be significantly more effective than the current existing training material for SCM Education (Table 3). The mean effectiveness of education material with and without the educational bundle was 7.77 (SD = 1.31) and 6.44 (SD = 1.98), respectively (p < 0.001). There were also significant improvements in ease of finding information on SCM (7.67 vs 7.14, p = 0.016), performing general functions (7.50 vs 6.89, p = 0.0050), and overall efficiency in SCM use (7.39 vs 6.92, p = 0.022).

**Table 3:** Comparisons between mean scores pre and post intervention SCM: Sunrise Clinical Manager, SDT: Specially Designed Test *Paired T-Test †2-Sample T-Test

Domain of Assessment	Pre Intervention (N = 36)	Post Intervention (N = 36)	P-value
	Mean Score ± SD	Mean Score ± SD	
Effectiveness of Education Material*	6.44 ± 1.98	7.78 ± 1.31	<0.001
Ease of Finding Information in SCM*	7.14 ± 1.66	7.67 ± 1.43	0.016
Ease of Performing Functions in SCM*	6.89 ± 1.70	7.50 ± 1.68	0.0050
Efficiency in using SCM*	6.92 ± 1.57	7.39 ± 1.29	0.022
Satisfaction in using SCM*	6.64 ± 2.07	7.06 ± 1.76	0.066
Scores on SDT (2 Arm Study)^†^	8.97 ± 1.83	9.33 ± 1.76	0.40

The mean score of how effective different education modalities rated in supplementing existing material is 7.19 for TikTok-style videos, 6.69 for Long-Form Videos (Wizlearn), and 6.75 for the Handbook. Although the authors did not find a statistical significance in effectiveness across various education modalities (p = 0.250), TikTok-style videos were the most well-received out of the three modalities based on the highest mean score attained.

Qualitative results

General comments were also collected from participants as part of the survey. These general comments were analyzed qualitatively for an overall response (positive, negative, or neutral) towards the intervention and if response was positive, which modality was highlighted. Overall positivity of the study would likely indicate the study being well received which will likely translate to better pickup of skill and knowledge.

**Figure 1:**
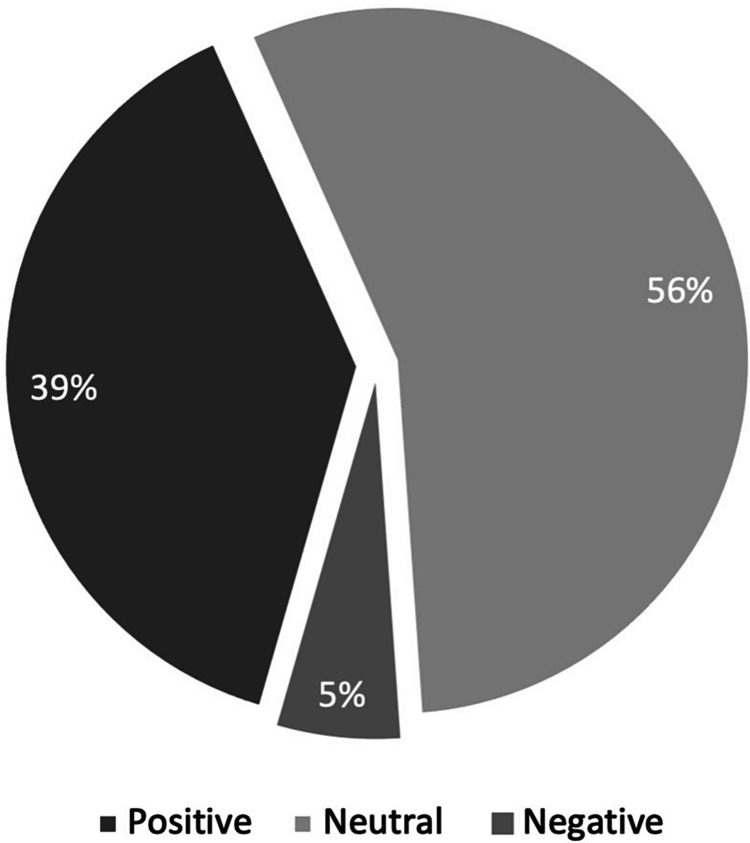
Pie chart representing general (positive, negative, or neutral) feedback of participants regarding intervention bundle administered

**Figure 2:**
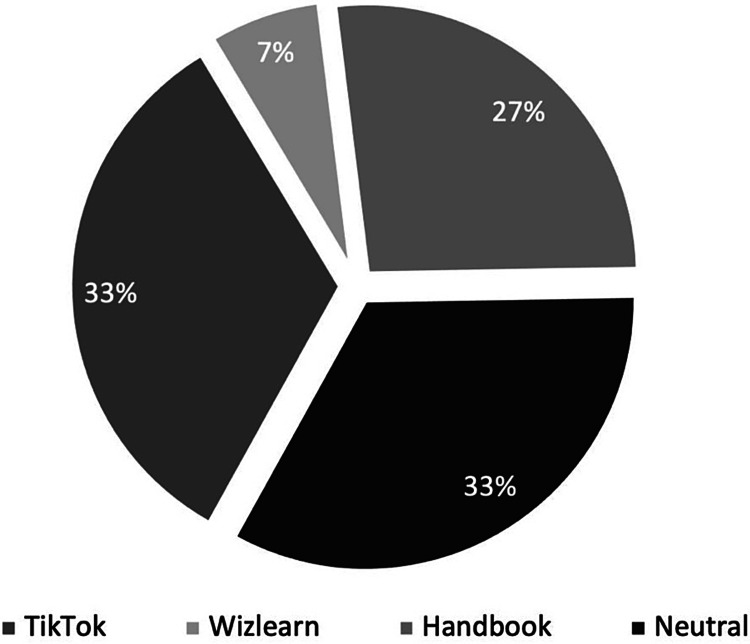
Breakdown of favorability of interventional bundle components in participants with positive feedback

More than a third of the study population found the interventional bundle to be a positive experience (38.89%), whereas only two participants (5.56%) found it to be negative (Figure 1). Those who did not leave any comments were marked as neutral (55.56%). Next, the subset of participants with positive feedback (n=14) was evaluated, and it was found that five (33.3%) highlighted TikTok videos, four (26.7%) highlighted the handbook and one (6.7%) highlighted the long-form video (Wizlearn) as their main cause of positivity. Those who did not highlight any modality were rated as neutral (Figure 2). For negative comments, one HO highlighted that TikTok-style videos were “too fast,” while another was unsure regarding the utility of the intervention bundle. Overall, comments for TikTok-style videos were positive for their ability to “entertain and catch attention” and being “concise and effective.”

## Discussion

The education bundle is novel through three main aspects. Firstly, it was designed by experienced end users (junior doctors for junior doctors). The authors believed that new users may find more value in training materials designed to cater to their daily clinical workflow with end users’ accumulated EHR knowledge and expertise, compared to those designed by traditional system designers. Involvement of end users in design is supported both by literature [11,12], and also by feedback gathered - “Personalized teachings such as from the QI project is much appreciated” and “Would love to be taught by colleagues if possible.” Secondly, this approach aims to supplement, rather than replace existing training material. This is useful to almost all existing institutions incorporating EHR into their healthcare delivery, as there is no need to alter existing practices to improve optimization. Thirdly, the education bundle leverages TikTok-style videos. One of the defining characteristics of the current generation of junior doctors, many of whom belong to Generation Z, is their familiarity and affinity for digital platforms [13]. Recognizing this trend, the authors integrated the use of TikTok-style videos as a novel and effective educational tool for imparting EHR knowledge to junior doctors.

At the heart of the education bundle is a series of TikTok-style videos, with the educational content being selected by the core group of five junior doctors and one consultant. This study's findings suggest that TikTok-style videos are non-inferior to other modalities, with no difference in effectiveness compared to more traditional educational modalities such as long-form videos and handbooks (p-value 0.250). To the authors' knowledge, TikTok-style videos have been used in other areas of medical education [14,15], but this study is the first to have utilized it for EHR optimization. This study's findings suggest that junior doctors are receptive to TikTok-style videos as a form of education. This may not be entirely surprising given the younger demographic of junior doctors, who are more familiar with and have a stronger affinity for digital platforms and social media [16]. In addition, the authors argue that TikTok-style videos may be especially fitting in the realm of EHR optimization, where tips and tricks are usually more bite-sized compared to medical concepts that often need a larger body of knowledge to appreciate.

One of the study’s challenges was the methodology to evaluate the effectiveness of EHR training materials. There remains a lack of existing literature for standardized methodology and evaluation models for EHR training [17,18]. A time-based approach was suggested by Sinsky et al. [19] to measure efficiency, where time spent on the EHR (both total and based on specified domains) was considered. However, the authors felt that this approach was not viable in determining the effectiveness of training material due to confounders. Firstly, how time spent on EHR is measured likely includes idle time, which varies from user to user. Secondly, measuring time spent also does not consider processing speed, which is affected by hardware and connectivity issues.

To this end, Kirkpatrick’s four-level training evaluation model was useful as a reference for assessing the intervention bundle [20]. Kirkpatrick’s four-level training evaluation model measured responses at four levels: (1) Reaction to Training, (2) Learning from Training, (3) Behavior Changes from Training, and (4) Organization Results. The self-administered questionnaires showed encouraging results for level one and level two. Firstly, more than a third of participants found the interventional bundle to be a positive experience (38.89%), compared to only 5.56% finding it to be negative. Secondly, in terms of learning, most measures showed improvement after the introduction of the education bundle, including gathering information on EHR and performing general functions.

For level three (Behavior Changes), the authors experimented with an approach involving an objective measure of applying their learning via a 12-question SDT. This is not a validated test and was an effort by the authors to represent a way of assessing how end users applied their knowledge as part of their work. While there was no statistical improvement in score after the intervention, the authors recognized that the SDT as a modality of testing is not previously validated and is, in fact, an experimental model. The authors wish to highlight that with a more comprehensive test with an increased number of data points, results could have been different, but it was difficult to design a test significantly more robust within the constraints of this study. The development of such a tool on its own may be an area of research for future studies.

Limitations

This is a small study that aimed to quickly assess the utility of an education bundle designed by end users, and the reception towards TikTok-style videos. Generalizability to all settings is challenging given that it is conducted within one institution. However, given that EHR systems are aplenty, and often modified to institutional requirements, it is the authors' opinion that a one-size-fits-all-education package is not possible. Instead, the aim of this study was to demonstrate that employing an end-user-driven education package in optimizing EHR knowledge is reasonable, and worth exploring by other institutions.

In addition, given that the sample is not large, one may wonder whether it is representative of junior doctor populations. One constraint the authors faced was being limited to testing only one cohort of HO, given the possibility of contamination effect. One measure to mitigate this was how selection bias was minimal, given that the study population of 36 HO represented more than 90% of new HO. On relevant demographic data such as educational background and prior exposure to SCM, there was no significant difference between groups as well (though this point is only relevant for the two-arm T-test for the SDT).

Lastly, the authors recognized that level four of Kirkpatrick's model (Organization Results) was not adequately explored. This is a key limitation of the study given that organization data required a much larger effort to collect and is worthy of a study on its own. However, given the low-cost approach to this study, areas such as cost savings and return on investment were likely going to be positive. On top of the positive results in the earlier three, the authors felt confident that their approach would reap positive benefits.

## Conclusions

This study has demonstrated how an end-user-driven educational bundle focusing on high-yield, advanced functions may be useful in enhancing the overall EHR system experience for junior doctors. Of note as well, is how TikTok-style videos seem no less effective than more traditional methods of EHR teaching. The hope is that the demonstrated approach can encourage other healthcare institutions to develop similar educational bundles to improve the overall EHR experience for junior doctors.

## Appendices

Below Appendices are available in PDF format on request:

A: SCM Handbook.pdf

B: Self-Administered Questionnaire.pdf

C: Specially Designed Test.pdf
